# Death Anxiety and Trauma in Forcibly Displaced Children in the Gaza Strip: A Critical Review of Emerging Research, 2024–2025

**DOI:** 10.1007/s11920-026-01670-8

**Published:** 2026-03-19

**Authors:** Anies Al-Hroub

**Affiliations:** 1 Department of Education, American University of Beirut, P.O. Box 11-0236, Beirut, Lebanon; 2 Fitzwilliam College, University of Cambridge, Visiting Fellow, Cambridge, UK

**Keywords:** Gaza, Children, Post-traumatic stress disorder (PTSD), Death anxiety, Grief, Resilience, Continuous traumatic stress, Decolonial psychology, War trauma

## Abstract

**Purpose of Review:**

This review synthesizes recent empirical and theoretical studies from 2024 to 2025 that examine the psychological effects of war, displacement, and siege on Palestinian children in the Gaza Strip. It focuses on the interconnected issues of death anxiety, post-traumatic stress disorder (PTSD), grief, and resilience, aiming to frame mental health outcomes within structural and political contexts rather than within individual pathology.

**Recent Findings:**

Studies indicate that between 70 and 90% of children in the Gaza Strip meet criteria for PTSD or exhibit significant symptoms of depression and anxiety. Continuous traumatic stress stems from prolonged bombardment, displacement, and loss, exacerbated by the collapse of mental health services. Qualitative evidence reveals widespread death anxiety, anticipatory grief, and moral injury. However, resilience rooted in family cohesion, faith, education, and Sumud (steadfastness) remains a key protective factor. Community-based interventions show potential but lack sustainability amid systemic destruction. Decolonial scholarship further challenges Western trauma models, treating children’s stories as acts of agency and epistemic resistance.

**Summary:**

The psychological impact of the military siege and sustained attacks in the Gaza Strip on children is profound and enduring. Effective responses should integrate psychosocial, educational, and justice-oriented approaches to rebuild community systems and explicitly acknowledge the political conditions that shape children’s lives. Future research should employ longitudinal, participatory, and culturally grounded methods that link psychological recovery to social reconstruction and the restoration of dignity.

## Introduction

For over fifteen years, the Gaza Strip has symbolized collective suffering, isolation, and resilience. Home to more than 2.3 million Palestinians, half of them children, Gaza has endured a continuous blockade since 2007, along with multiple military attacks that have destroyed infrastructure, undermined livelihoods, and shattered every aspect of daily life [1].

During periods of intensified siege and military attacks on Gaza, including 2008–09, 2012, 2014, 2021, and the unprecedented, systematic destruction of civilian, educational, and cultural infrastructure since October 2023, schools became shelters and teachers acted as first responders. Between October 2023 and July 2025, Gaza’s formal education system nearly collapsed. Official figures indicate that over 17,000 Palestinian students, including 16,124 school students and 1,191 university students, were killed during this period. Of these, 16,019 school students and 1,156 university students died inside Gaza. Meanwhile, 24,614 school students and 21,191 university students were injured, with 23,913 and 2,351 of these injuries occurring in Gaza. Additionally, 88,000 university students in Gaza have been unable to continue their studies because all universities in the territory have remained closed since hostilities started. Approximately 39,000 Grade 12 students have missed the General Secondary Examination (Tawjihi) for two consecutive cycles because examination centers and schools were rendered inoperable.

This widespread loss of life, along with the destruction of 90% of Gaza's schools [2], places children's trauma at the heart of an unprecedented humanitarian moral crisis. This relentless violence has turned Gaza into what Veronese et al. describe as a “continuum of trauma,” in which recovery is impossible because the threat endures. Continuous bombings, deprivation, and exposure to death have led to alarming rates of psychological disorders [2]. Studies consistently show that between 70 and 90% of children exhibit symptoms of post-traumatic stress disorder (PTSD), depression, or severe anxiety [3, 4]. The psychosocial effects extend beyond diagnoses to include existential fear, death anxiety, anticipatory grief, and a loss of meaning [5].

## Purpose and Scope of the Critical Review

This critical review synthesizes and evaluates the growing body of research on death anxiety, PTSD, grief, and broader mental health outcomes among forcibly displaced Palestinian children in Gaza. A total of 15 empirical studies, two narrative reviews, and one systematic review published between 2024 and 2025 were examined. These studies employ a wide range of methodologies, including qualitative interviews [5, 6], large-scale quantitative surveys [3, 7], psychometric assessments using standardized trauma instruments [4, 7], machine learning–assisted analyses [8], longitudinal designs [2], community-based intervention evaluations [9], and a systematic review synthesizing prior research [10]. Additional contributions include content analyses of children’s war testimonies [11] and narrative studies [12] focusing on children and young adolescents. These empirical findings are further contextualized by a systematic review [10] that highlights structural and infrastructural barriers to mental health service delivery during war.

The aims align with the review's thematic organization. Specifically, it examines: (a) the prevalence, types, and phenomenology of trauma-related distress among Gazan children; (b) the sociopolitical, ecological, and structural factors that produce and maintain their mental health outcomes; (c) the role of education, family, community, and cultural values in fostering resilience and meaning-making; and (d) the limitations of humanitarian and psychological responses grounded in Western, individual-centered frameworks that fail to capture the collective and political aspects of Palestinian suffering fully.

## Continuous Traumatic Stress and the Ecology of War

The psychological state of Gaza’s children cannot be understood through trauma models based on isolated, time-limited events. Instead, the evidence overwhelmingly supports the continuous traumatic stress (CTS) framework, a condition characterized by ongoing, predictable, and inescapable danger. Veronese et al. argue that CTS more accurately reflects the experiences of children exposed to relentless bombardment, displacement, and deprivation, with no time for healing or processing [2]. Peltonen’s developmental analysis reinforces this, showing how chronic insecurity influences neurocognitive development by constantly activating fear circuits and hindering the formation of stable attachments [13].

This ecological perspective is reinforced by Farajallah, who describes Gaza as a closed trauma field. The destruction of infrastructure further exacerbates this situation. Qutishat reports that the destruction of hospitals, schools, and mental health centers has eliminated support and containment options, leaving children without protective social networks [10].

Notably, CTS in Gaza is not only psychological but also geopolitical. Farajallah explains that the blockade itself acts as a constant stressor through deprivation, forced immobility, and ongoing surveillance (such as drones), fostering a continual sense of danger even during ceasefires [14]. Peltonen also points out that ongoing threats alter children’s mental frameworks: they come to expect violence as usual, displaying hypervigilance even during brief periods of calm [13].

These insights place Gaza’s trauma beyond simple clinical categories, framing it within a broader structural ecology of violence. CTS is the primary model for understanding the psychopathology associated with exposure, including PTSD, depression, anxiety, and behavioral problems.

In line with the CTS and structural violence framework, Schöler et al. conducted a large cross-sectional, school-based survey of 11,646 first-grade Palestinian refugee children (mean age = 6.1 years) across 135 United Nations Relief and Works Agency for Palestine Refugees in the Near East (UNRWA) schools in all five Gaza Strip governorates, following the May 2021 Israeli war on Gaza. Using a convenience sample with near-population coverage, the study found that 75.9% of children reported direct or subjective exposure to violence. Trauma symptoms increased in a graded manner across exposure levels and peaked among those experiencing both. Symptom severity varied by governorate and was attenuated among higher-income families. Functional impairment affected 38.1% of children and increased with exposure severity, while elevated trauma symptoms persisted regardless of assessment timing [7].

## Trauma-Related Psychopathology and PTSD Symptoms

Within Gaza’s ecology of ongoing threat, post-traumatic stress symptoms and related trauma-related psychopathology are highly prevalent among children [2, 3, 7, 8]. Large cross-sectional studies show pervasive distress following repeated military assaults; for example, Aldabbour et al. report that nearly all young adults exposed to the Gaza war exhibited symptoms of depression (97.1%), anxiety (84.4%), and stress (90.6%), with a substantial proportion also reporting PTSD symptoms [3].

Trauma exposure in Gaza also operates intergenerationally. Ashour and Graham demonstrate that parental fear, helplessness, and survivor's guilt shape children’s emotional environments, positioning families as both recipients and transmitters of distress [4]. At the neurocognitive level, Manzanero et al. distinguish between socially preserved and socially weakened trauma profiles in preadolescents, illustrating how chronic fear and relational disruption influence the expression and severity of PTSD-related symptoms [8]. Broader reviews and conceptual syntheses further suggest that parental warmth, religious meaning, and collective identity may partially buffer symptom severity, though these protective factors cannot offset continuous exposure to violence [13, 14].

Physiological manifestations reinforce this pattern of chronic trauma. Hamamra et al. describe persistent insomnia, nightmares, and hyperarousal among displaced women and children as indicators of ongoing existential insecurity rather than time-limited post-traumatic reactions [5]. Vulnerability is further compounded among children with disabilities and those forced into premature adult roles, who face heightened exposure with fewer developmental and protective resources [15].

Overall, the evidence positions PTSD symptoms in Gaza not as isolated clinical outcomes but as manifestations of chronic traumatic stress embedded in prolonged siege and the structural realities of blockade, displacement, and existential insecurity, necessitating a shift from post-traumatic to continuous-trauma frameworks.

## Death Anxiety, Anticipatory Grief, and Existential Fear

In a setting where death is omnipresent, Gaza’s children experience trauma not only from fear of harm but also from constant exposure to mortality. Hamamra et al. provide strong evidence of this, showing widespread death anxiety among children who frequently witness deaths, anticipate further losses, and feel survivor's guilt. Their findings reveal a mental landscape in which children ask unusual questions for their age, such as “Why am I alive?” and “Who will die next?”, indicating they are internalizing existential threats as part of their daily thoughts [16]. Farajallah broadens the concept of death anxiety to include psychological fear, moral injury, children’s guilt for surviving, feelings of helplessness in protecting loved ones, and the unraveling of beliefs about fairness and safety [14]. He describes a society in “anticipatory mourning,” where grief is continuous and collective, worsened by the destruction of cemeteries and limited burial rituals that prevent families from finding closure. This ongoing trauma spills into schools, creating what Veronese et al. call an “educational echo chamber of grief,” where learning becomes both a means of survival and a shared space of mourning [17]. Affouneh et al. and Jawad et al. show that children interpret these losses through a moral–political lens, viewing them not only as personal tragedies but also as violations of dignity [11, 18]. Their narratives often invoke justice, shahada (martyrdom), and collective memory to make sense of mass death. Despite these existential pressures, children actively sought meaning through everyday practices and imaginaries that reaffirm belonging and continuity; they maintained affective ties to place (the sea, the land, the home), transformed disrupted environments into sources of reassurance, and projected themselves into imagined futures grounded in family, community, and generational histories, rather than remaining confined to frames of trauma alone [18].

Hamamra et al. and Jawad et al. show that children use testimony, storytelling, and drawing not only to express distress but also to reclaim agency in how their experiences are understood. These practices constitute what Jawad terms epistemic resistance: children actively producing their own explanations of violence, such as naming injustice, affirming belonging to land and community, and situating loss within collective and historical frameworks, rather than allowing their suffering to be interpreted solely through depoliticized clinical or humanitarian narratives. For example, children insist on attachment to the sea or land as sources of identity, rework everyday objects such as seashells into forms of reassurance and memory, and articulate future-oriented hopes grounded in family and generational continuity. Religious meaning similarly plays a dual role, sometimes buffering fear and at other times intensifying grief, underscoring the need for culturally grounded analysis rather than assumptions of uniform protective effects [16, 18].

Overall, death anxiety in Gaza cannot be reduced to a pathological fear. It reflects a broader condition of existential siege, a mental state shaped by ongoing exposure to death, disrupted mourning, moral confusion, and systemic violence. Recognizing this is key to understanding how trauma shifts from fear of harm to a lasting confrontation with annihilation and injustice.

## Childhood, Education, and the Loss of Rights

The prolonged siege has transformed schools, once safe spaces of security and identity, into ruins or temporary shelters. For children in Gaza, education symbolizes both a memory of normality and a fragile hope for continuity amid destruction. Affouneh et al. note that these children view the right to education as inseparable from the right to life itself [11]. Their participatory study, centered on children's perspectives, reveals that even during bombings, many wish to return to school as an act of resistance and dignity. Bdier et al. [6] examine how education serves as both a source and a victim of resilience. During the 2023–2025 military attack on Gaza, students described studying amid rubble and displacement as a way to assert their personhood against dehumanization. For them, learning is not only cognitive but also existential; it is a means of maintaining a sense of time, purpose, and moral order. They call this the pedagogies of steadfastness, in which learning serves as a psychological shield against despair. This aligns with Veronese et al., who argue that educational spaces in war zones function as psychosocial ecosystems that nurture hope and relationships despite interruptions to formal instruction [2].

Ashour and Graham note that parents’ perspectives highlight the psychological toll of disrupted education [4]. Parents reported that the destruction of schools and the halt to classes caused deep anxiety in children, stemming not only from academic setbacks but also from the breakdown of daily routines, friendships, and safety rituals. Interviews indicate that children are beginning to “unlearn” joy and curiosity, associating books and uniforms with memories of loss. This symbolizes a breakdown: education no longer serves as a developmental bridge but as a reminder of systemic abandonment. Beyond immediate trauma, this destruction has lasting psychological effects. Bdier et al. emphasize that displacement forced many children to study in overcrowded shelters or informal spaces, where limited space, lack of privacy, and the threat of airstrikes hinder concentration and learning [6].

The absence of safe learning environments exacerbates cognitive overload and feelings of futility. Teachers and many grieving family members face burnout and survivor's guilt, which impair their ability to provide emotional support. Disparities are intensified by gender, disability, and displacement; girls and children with disabilities encounter greater barriers to accessing what remains of schooling. Bdier et al. and Farajallah state that these children are often excluded from emergency learning spaces due to physical inaccessibility, social stigma, and resource prioritization for boys [6, 14]. Such exclusion perpetuates structural discrimination and deepens psychological isolation and self-doubt. Education, once a tool for empowerment, now also represents marginalization. The loss of education in Gaza goes beyond academic deprivation; it signifies the breakdown of childhood as a protected life stage. Farajallah describes this as the “stolen temporality of Gaza’s youth,” where time feels suspended between destruction and anticipation of further loss. Nevertheless, acts of learning continue as subtle acts of resistance [14]. Affouneh et al. and Bdier et al. describe children rebuilding informal classrooms, teaching peers, or writing messages of hope on debris [6, 11].

These small acts, such as reading by candlelight, sharing notebooks, and reciting poetry in shelters, embody what Palestinian educators call sumud al-ta ‘lim (steadfast learning). In this way, education becomes a psychosocial lifeline, sustaining the belief that knowledge and moral awareness persist even when institutions collapse.

## Resilience, Faith, and the Communal Ethic of Sumud

Research indicates that children in Gaza rely on community, cultural, and spiritual resources to sustain psychological well-being amid ongoing hardships [2, 13]. In this context, resilience cannot be understood through individualistic or trait-based models typical of Western psychology. Instead, Peltonen and Veronese et al. propose an ecological framework in which resilience arises from interdependence, family cohesion, collective identity, spiritual significance, and community networks [2, 13]. Their findings suggest that shared routines, a sense of belonging, and culturally rooted stories confer protective effects that exceed those of individual coping strategies. However, the cross-sectional design of their studies limits causal inference. Intervention evidence supports this ecological perspective. D’Andrea et al. evaluated The Eye to the Future, an extensive community-based psychosocial program, and observed significant reductions in children’s emotional and behavioral problems, along with improvements in social connections and a more positive outlook on the future [9]. These outcomes are attributed to the program’s community-led approach, local mentors, group activities, and culturally embedded practices rather than formal clinical therapy. However, limited follow-up data restrict conclusions about the long-term sustainability of these effects [9].

Cultural practices also play a vital role in fostering resilience. Humor, storytelling, religious rituals, and shared narratives serve as culturally embedded mechanisms that help preserve continuity and coherence when formal support systems are absent [14, 17]. These practices align with Peltonen's description of adaptive communal resilience. They also reveal potential risks, such as emotional suppression or normative pressure to endure, which reflect defensive resilience [13]. Further evidence indicates that access to communal resilience resources is unequal. Bdier et al. report that children with disabilities often face barriers to participating in communal coping structures due to infrastructure issues and social stigma [6]. Similarly, Hamamra and Shehab describe processes of unchilding, in which children assume adult responsibilities during socioeconomic crises, adding extra stress to family and community networks that usually support coping and emotional regulation [15].

Collectively, these studies view resilience in Gaza as a socially and culturally rooted process rather than an individual psychological trait [2, 13]. The concept of sumud, meaning steadfastness, captures this collective view, highlighting the importance of shared meaning-making, community care, and cultural practices in maintaining function during a prolonged crisis. At the same time, Qutishat warns that resilience analyses must remain aware of the material and political factors shaping these processes, as focusing only on adaptive outcomes can obscure the structural violence that drives the need for resilience [10].

## Decolonizing Trauma Narratives and Children’s Agency

A growing body of decolonial scholarship challenges the assumption that Western trauma models can be universally applied in Gaza, noting that clinical categories alone do not fully capture the political, moral, and historical factors shaping children’s experiences [14]. Jawad et al. critique humanitarian frameworks that cast children as passive victims in need of therapeutic correction. Their narrative analyses show that children describe displacement, loss, and daily survival in ways that affirm identity, belonging, and moral understanding. These testimonies constitute forms of epistemic agency, emerging in a context where more than 95% of children have been displaced, and over 70% have lost a family member by late 2024 [18].

Peltonen’s developmental–ecological approach reinforces this critique. He shows that Western measures of adaptation, typically focused on individual functioning, do not fully capture children’s moral reasoning, collective responsibility, or relational coping strategies in the context of chronic threat. His findings underscore the need for frameworks that consider sociopolitical context and collective meaning-making [13]. This critique is further supported by Veronese et al. [2, 17], whose mixed-methods studies examine how Gazan children understand and cope with ongoing violence. Their quantitative results reveal patterns that diverge from those expected in Western trauma models, including moderate life-satisfaction scores despite widespread trauma exposure and declines in perceived personal agency alongside stable or strong community belonging. These findings suggest that coping and well-being are influenced by collective identity, relational bonds, and political context rather than individual indicators alone. Qualitative data further show that children interpret distress, hope, and daily functioning through cultural, relational, and sociopolitical lenses [19].

Together, this evidence suggests that psychological outcomes in Gaza must be understood within culturally and politically grounded frameworks. Farajallah similarly warns that overreliance on diagnostic categories can obscure the structural and colonial conditions that cause distress [14]. Building on this, Qutishat argues that mental health responses in Gaza require decolonized policy approaches, including local governance, community involvement, and alignment with human rights frameworks, because many psychosocial programs remain externally designed and lack sustainability [10]. These perspectives collectively call for a paradigm shift: from viewing Gazan children primarily as traumatized patients to seeing them as moral witnesses, cultural narrators, and political agents. Decolonizing trauma studies, therefore, entails combining quantitative indicators of harm with detailed qualitative accounts of children’s lived experiences, thereby respecting both the extent of suffering and their agency in interpreting and resisting it.

## Conclusion

A critical review of the literature highlights a clear and urgent insight: Gaza’s children's psychological health is best understood as the result of ongoing traumatic stress rooted in a collapsing social environment, rather than isolated traumatic events. While children’s experiences vary according to age, gender, disability, and family circumstances, across quantitative, qualitative, and theoretical studies, a consistent pattern emerges that fundamentally challenges traditional trauma models.

First, the evidence shows extremely high levels of PTSD, depression, anxiety, and death anxiety. These conditions stem from an environment where violence is frequent, displacement is standard, and family stability is disrupted. Trauma is thus not merely a psychological reaction but an expected outcome of living under siege, infrastructural damage, and persistent insecurity.

Second, the literature reveals severe disruptions to childhood development. Loss of educational continuity, disruption of daily routines, and the phenomenon of “unchilding” demonstrate how war undermines the foundations of play, learning, attachment, and imagination. Third, research emphasizes coping strategies rooted in community, culture, and shared meaning-making. However, findings caution against romanticizing resilience: while community-based efforts lead to measurable improvements, they cannot replace the broader structural protections that are missing. Fourth, adopting a decolonial perspective is crucial for understanding children’s experiences. Children’s stories, testimonies, and creative expressions challenge the idea that they are passive victims. Instead, they act as moral witnesses, interpreting their experiences through the lenses of justice, belonging, memory, and resistance. This shift in perspective urges trauma research to recognize the political and historical forces behind suffering rather than reducing distress to clinical symptoms disconnected from context.

Finally, Gaza’s near-total collapse of mental health services leaves children with almost no access to formal psychological support. The destruction of clinics, schools, and community centers has eradicated key environments for recognizing and addressing distress. In these circumstances, individual treatment alone is unlikely to be effective; mental health responses must address the structural barriers to care. Collectively, these studies call for a fundamental change in global trauma research, one that recognizes the moral, political, and historical roots of suffering. Gaza’s children, despite profound loss, continue to show resilience, creativity, and perseverance. Supporting their healing extends beyond clinical need and raises profound moral obligations, requiring justice, reconstruction, and the restoration of dignity. Only by addressing both the psychological and structural wounds of war can future generations of Gazan children move from merely surviving to truly thriving in peace.

Nevertheless, while the literature consistently identifies structural and chronic trauma as defining features of children’s psychological distress in Gaza, these conclusions must be interpreted in light of significant methodological constraints, including restricted research access, disrupted data collection, and reliance on crisis-context and NGO-led studies.

## Directions for Future Research

Future research on the mental health of Gaza’s forcibly displaced children should move beyond basic epidemiology and focus on approaches that are contextually grounded, ethically engaged, and longitudinal. Long-term studies are essential for understanding how ongoing trauma affects development across generations and how chronic stress influences emotion regulation, cognition, and family relationships. Such research would benefit from integrating biological, psychological, and sociocultural data to build detailed models of adaptation.

Further research is needed to examine how disrupted schooling and the loss of safe, structured environments affect development. Studies should investigate how alternative learning methods, such as informal peer networks, community-based study groups, and caregiver-led instruction, function when formal education systems collapse, and whether these methods help restore consistency and stability in children’s daily lives.

Intervention research should also focus on locally driven, culturally relevant models. Community programs such as The Eye to the Future [9] show that support systems grounded in collective effort and cultural significance can be effective. However, little is known about which elements enhance their sustainability. Future evaluations should examine mechanisms of change, barriers to continuity, and factors that enable local practitioners, rather than outsiders, to lead and sustain psychosocial interventions.

Methodologically, future research should prioritize children’s perspectives. As Affouneh et al. and Jawad et al. show, narrative, artistic, and ethnographic methods reveal aspects of experience that standard clinical tools often overlook [11, 18]. Expanding these approaches could deepen understanding of how children interpret loss, maintain hope, and find moral meaning amid ongoing violence.

There is also a need for research that accounts for within-child variation. Intersectional analyses focusing on gender, disability, poverty, and orphanhood are crucial for understanding diverse vulnerabilities and for designing interventions tailored to specific needs, rather than treating children as a uniform group.

Finally, future research should incorporate decolonial and moral-psychological frameworks. Psychological recovery should not be limited to symptom reduction while structural violence persists. Drawing on liberation psychology and moral injury theories can help researchers examine how justice, dignity, and collective memory function as vital elements of healing.

##  Key References

- Veronese G, Bdier D, Obaid H, Mahamid F, Yaghi S, Cavazzoni F. Agency, life satisfaction, hope, potentially traumatic events, trauma symptoms, and psychological signs. A two waves study with a sample of Palestinian children living in different geographical areas. Children and Youth Services Review. 2025 Jan 1; 168:107990.
  - ○ This study provides rare empirical evidence on how ongoing warfare and settler-colonial violence affect children’s psychological well-being, sense of agency, and hope—key areas for healthy development. Analysing data from 965 Palestinian children, including 494 males and 471 females across two waves in four regions **(**East Jerusalem, West Bank, and the Gaza Strip) and contexts (urban, rural, and refugee camps), shows rising trauma symptoms and decreasing agentic resources, indicating increased vulnerability over time. The study emphasizes region-, gender-, and age-specific risk patterns, offering vital data for designing targeted mental health, protection, and educational programs in high-risk areas like Gaza, Jerusalem, and Bedouin communities.
- Schöler N, Gal G, Wissow LS, Seita A. Stress and Trauma Symptoms in Young Palestine Refugee Children Following the May 2021 Escalation in Gaza. JAACAP Open. 2024 Aug 22.
  - ○ This large-scale study of 11,646 Palestinian refugee children aged 5–7 across all five Gaza Strip governorates reveals a clear trend: as children’s exposure to violence and fear levels rise, their trauma symptoms and functional difficulties also increase.
- Manzanero AL, Aroztegui J, Fernández J, Guarch-Rubio M, Álvarez MÁ, El-Astal S, Hemaid F. War, torture and trauma in preadolescents from Gaza Strip. Two different modalities of PTSD. Anuario de psicología jurídica. 2024;34(1): 1–2.
  - ○ This is a large-scale study aimed at assessing the impact of past traumatic war experiences on 521 preadolescents attending United Nations Relief and Works Agency for Palestine Refugees in the Near East (UNRWA) schools.
- Hamamra B, Mahamid F, Bdier D, Atiya M. War-related trauma in narratives of Gazans: challenges, difficulties and survival coping. Cambridge Prisms: Global Mental Health. 2025 Jan;12:e34.
  - ○ This qualitative study offers one of the most detailed accounts of pervasive death anxiety and disrupted mourning among Palestinians during the 2023–2024 genocide. It is crucial for your review because it links existential fear, grief, and cultural erasure to concrete war practices (e.g., denial of burial), while also documenting religious and familial sources of resilience.
- Bdier D, Hamamra B, Mahamid F. From laughter to survival: The effect of war on children’s play in Gaza. Children and Youth Services Review. 2025 Aug 9:108526.
  - ○ This qualitative study foregrounds disabled children and their caregivers, revealing catastrophic regression, loss of services, and extreme caregiver strain.
- Veronese G, Mahamid F, Obaid H, Bdier D, Cavazzoni F. Positive and negative effects of child’s agency on trauma symptoms and psychological difficulties in war-like conditions. The mediating role of hope and life satisfaction. Journal of Mental Health. 2024 Nov 1;33(6):749-58.
  - ○ A large-scale study examined the connection between agency, trauma symptoms, and psychological issues, and the mediating role of hope and life satisfaction in nine hundred sixty-five children aged 8 to 14, who were victims of military violence in Palestine.

## Data Availability

In this review article, no new data were created or analyzed. Data sharing does not apply to this article.
